# The Ubiquitin Sensor and Adaptor Protein p62 Mediates Signal Transduction of a Viral Oncogenic Pathway

**DOI:** 10.1128/mBio.01097-21

**Published:** 2021-09-07

**Authors:** Ling Wang, Mary E. A. Howell, Ayrianna Sparks-Wallace, Juan Zhao, Culton R. Hensley, Camri A. Nicksic, Shanna R. Horne, Kaylea B. Mohr, Jonathan P. Moorman, Zhi Q. Yao, Shunbin Ning

**Affiliations:** a Department of Internal Medicine, Quillen College of Medicine, East Tennessee State University, Johnson City, Tennessee, USA; b Center of Excellence for Inflammation, Infectious Diseases and Immunity, Quillen College of Medicine, East Tennessee State University, Johnson City, Tennessee, USA; c HCV/HIV Program, James H Quillen VA Medical Center, Johnson City, Tennessee, USA; University of North Carolina, Chapel Hill

**Keywords:** p62, EBV, LMP1, ubiquitination, viral oncogenesis, herpesviruses, p62

## Abstract

The Epstein-Barr virus (EBV) protein LMP1 serves as a paradigm that engages complicated ubiquitination-mediated mechanisms to activate multiple transcription factors. p62 is a ubiquitin sensor and a signal-transducing adaptor that has multiple functions in diverse contexts. However, the interaction between p62 and oncogenic viruses is poorly understood. We recently reported a crucial role for p62 in oncovirus-mediated oxidative stress by acting as a selective autophagy receptor. In this following pursuit, we further discovered that p62 is upregulated in EBV type 3 compared to type 1 latency, with a significant contribution from NF-κB and AP1 activities downstream of LMP1 signaling. In turn, p62 participates in LMP1 signal transduction through its interaction with TRAF6, promoting TRAF6 ubiquitination and activation. As expected, short hairpin RNA (shRNA)-mediated knockdown (KD) of p62 transcripts reduces LMP1-TRAF6 interaction and TRAF6 ubiquitination, as well as p65 nuclear translocation, which was assessed by Amnis imaging flow cytometry. Strikingly, LMP1-stimulated NF-κB, AP1, and Akt activities are all markedly reduced in p62^−/−^ mouse embryo fibroblasts (MEFs) and in EBV-negative Burkitt’s lymphoma (BL) cell lines with CRISPR-mediated knockout (KO) of the p62-encoding gene. However, EBV-positive BL cell lines (type 3 latency) with CRISPR-mediated KO of the p62-encoding gene failed to survive. In consequence, shRNA-mediated p62 KD impairs the ability of LMP1 to regulate its target gene expression, promotes etoposide-induced apoptosis, and reduces the proliferation of lymphoblastic cell lines (LCLs). These important findings have revealed a previously unrecognized novel role for p62 in EBV latency and oncogenesis, which advances our understanding of the mechanism underlying virus-mediated oncogenesis.

## INTRODUCTION

Constitutive NF-κB activation plays a decisive role in oncogenesis caused by several viral proteins, including Epstein-Barr virus (EBV) latent membrane protein 1 (LMP1), Kaposi's sarcoma-associated herpesvirus (KSHV) vFLIP, and human T-cell leukemia virus type 1 (HTLV-1) Tax. The signaling pathways triggered by these viral oncoproteins are complicated and still not fully understood. They share a large spectrum of components, including the involvement of the host ubiquitin machinery, but each may involve unique molecules (1–5).

The EBV principal oncoprotein LMP1 is required for EBV transformation and pro-oncogenic cell proliferation in multiple cell backgrounds as well as in transgenic mice (6), and is essential for the survival of EBV-transformed cells *in vitro*. As a pleiotropic factor, LMP1 plays crucial roles throughout the EBV life cycle, including lytic infection, reactivation, and latency, by reprogramming diverse cellular functions, such as metastasis, proliferation, apoptotic resistance, and immune modulation (7–11). Other EBV products, however, play complementary roles in cell proliferation and survival (12, 13).

The host ubiquitin machinery plays crucial roles in the host-pathogen interaction, highlighted by its intimate relation with oncogenic viral latency. Ubiquitination and similar modifications represent a pervasive theme important to the activation of NF-κB and other transcription factors in myriad processes, such as LMP1/Tax-mediated signal transduction (14–19). We showed previously that TRAF6/RIP1-dependent K63-linked polyubiquitination of IRF7 is required for its activation downstream of LMP1 signaling, whereas A20 acts as a deubiquitinase to negatively regulate LMP1-stimulated IRF7 activity (20–22). More recently, we showed that the linear ubiquitin assembly complex (LUBAC)-mediated linear ubiquitination modulates LMP1 signal transduction to NF-κB and IRF7 activation (23). These original findings provided novel mechanistic insights into LMP1 signal transduction.

p62 (also named EBIAP, ZIP3, and SQSTM1 [sequestosome-1]), a well-known selective autophagy receptor, also plays at least two ubiquitination-mediated roles in activating NF-κB in distinct contexts (24, 25). First, p62 has a ubiquitin-binding region (UBA) that enables its function as a “ubiquitin sensor,” which binds to K63 ubiquitin chains of ubiquitinated signal intermediators to facilitate IKKβ phosphorylation. Second, p62, as an adaptor protein, has a TRAF6-binding domain and specifically interacts with TRAF6, but not with TRAF5 or TRAF2, to facilitate TRAF6 K63-linked ubiquitination, in which both the N-terminal dimerization domain and the UBA domain are also required.

p62 plays oncogenic roles in different contexts, underscored by the facts that (i) its upregulation has been detected in several types of cancer, and (ii) it is induced by the oncoprotein Ras, which underlies at least 25% of human cancers (26–29). We showed recently that p62 mRNA and protein levels are in correlation with EBV latency programs, in which p62 mediates reactive oxygen species (ROS)-induced autophagy and DNA damage response (30). Two previous high-throughput screens identified p62 interaction with both the EBV-induced protein EBI3 and TRAF1 in EBV latency (31, 32). A recent report also showed that p62 is a component of the HTLV-1 Tax signalosome (33). The host ubiquitin machinery and TRAF6 are pivotal for LMP1 signal transduction. However, the mechanisms underlying p62 deregulation in EBV latency and whether its ubiquitin sensor and TRAF6-binding roles are involved in LMP1 signal transduction remain unknown.

In this study, we show that p62 is induced by NF-κB/AP1 downstream of LMP1 signaling in EBV latency. More importantly, p62 participates in LMP1 signal transduction in a positive feedback loop through its interaction with TRAF6 and contributes to LMP1 oncogenic functions.

## RESULTS

### LMP1 activates the p62 gene promoter via the NF-κB and AP1 axes.

**FIG 1:**
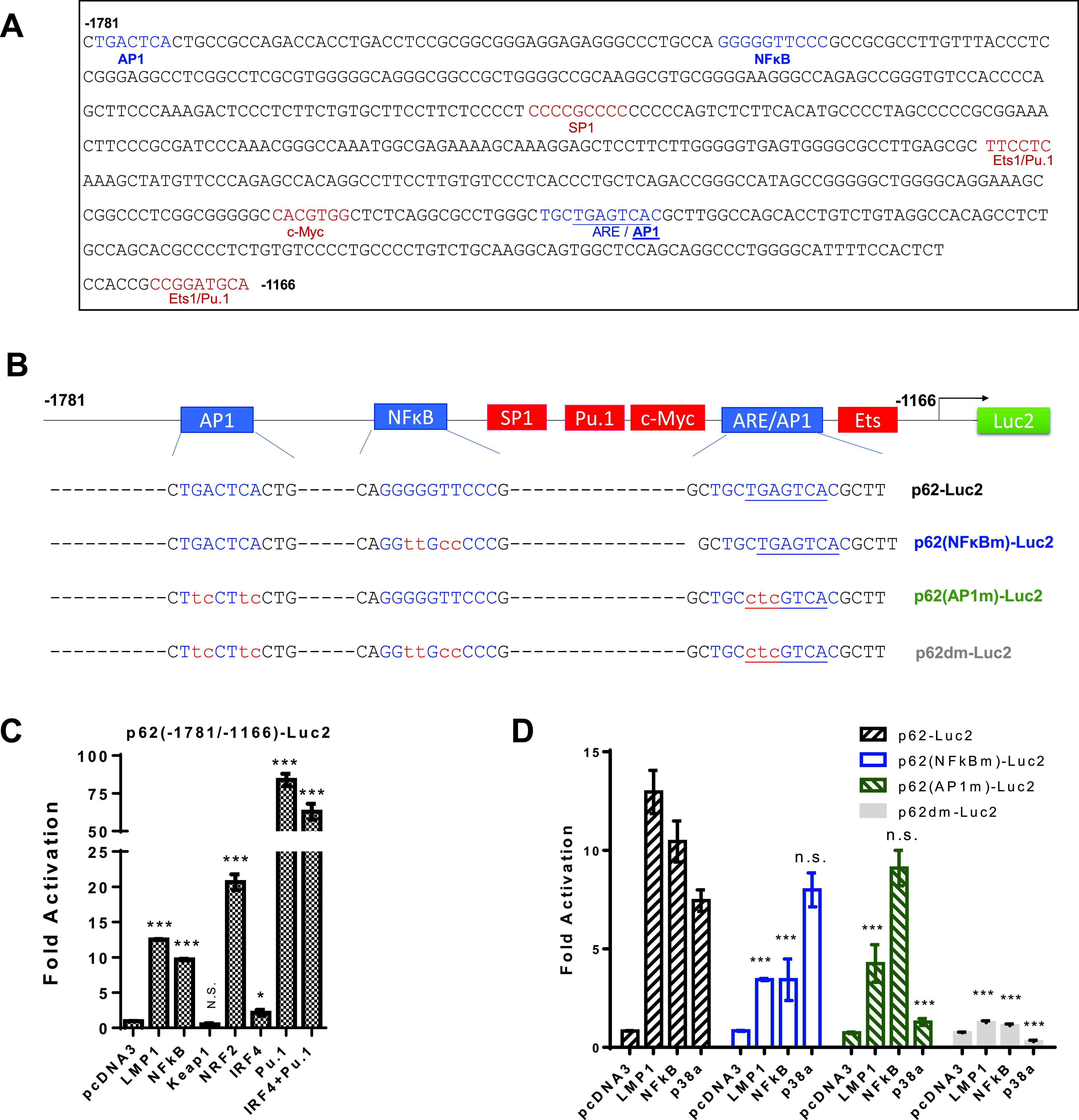
LMP1 activates the p62 gene promoter. (A) Transcription factor-binding sites on the promoter sequence spanning −1166 to −1781 of the human p62 gene. (B) Diagram showing the p62 promoter construct pGL3/p62(−1781/−1166)-Luc2 and its mutants. (C) LMP1, NF-κB, NRF2, and Pu.1 transactivate wild-type pGL3/p62(−1781/−1166)-Luc2. (D) Response of pGL3/p62(−1781/−1166)-Luc2 mutants to LMP1, NF-κB, and p38α. 293 cells in 24-well plates were transfected with 150 ng IRF4, NRF2, or Pu.1 and 150 ng p65 plus p50 (75 ng each), or 10 ng LMP1 and 40 ng pGL3/p62(−1781/−1166)-Luc2 or its mutants, and 10 ng *Renilla* luciferase, for duplicates. A dual-luciferase assay was performed 24 h after transfection. Consistent results were obtained from at least three independent repeats, and representative results are shown. The ability of the vector control to activate the promoter construct was set to 1. The statistical analysis was performed on the results to compare results for the wild-type promoter to those for the mutants. *, *P* < 0.05; ***, *P* < 0.01; n.s., not significant.

The p62 promoter (2.0 kb upstream of the transcription start site [TSS]) contains well-characterized NF-κB and AP1 binding sites, among others (Fig. 1A), and is activated by NF-κB and AP1 in diverse contexts (26, 27, 34–36). Furthermore, in our recent study, we unexpectedly found that short hairpin RNA (shRNA)-mediated deficiency of LIMD1, an adaptor protein required for LMP1 signal transduction to NF-κB and AP1 activation, reduces p62 expression in virus-transformed cells (37). We were thus motivated to investigate whether p62 is regulated by the LMP1-NF-κB/AP1 axes and plays a role in EBV latency.

We first investigated whether LMP1 can activate the p62 promoter through NF-κB and AP1 signaling axes. The human p62(−1781/+46)-Luc2 construct was excised to reduce high background in promoter-reporter assays in our system, resulting in a fragment spanning −1781 to −1166, which includes the known NF-κB- and AP1-binding sites (Fig. 1B), and this fragment was then subcloned into pGL4.23-miniP-Luc2. We also made a panel of mutants with either the NF-κB- or AP1-binding site mutated or both mutated, using site-directed mutagenesis (Fig. 1B). Note that the second AP1 site overlaps the antioxidant response element (ARE), which is responsible for NRF2 activation in antioxidant defense, and we mutated the AP1 core sequence but retained the ARE consensus binding motif (5′-RTGAYNNNGCR-3′; reversed in the p62 promoter) (Fig. 1B). A promoter-reporter assay was performed as described in many of our publications (21, 22, 38, 39). Results show that transfection of LMP1, NF-κB (p65+p50), NRF2, p38, or Pu.1 expression plasmids activates p62(−1781/−1166)-Luc2, but mutation of the core sequences of NF-κB- or AP1-binding sites dramatically reduces the p62 promoter activity in response to LMP1, NF-κB, or p38α (upstream of AP1) (Fig. 1C and D). These results indicate that LMP1, NF-κB, and AP1 can transactivate the p62 promoter.

The binding of NF-κB and AP1 to the p62 gene promoter was also detected by algorithm analysis (https://chip-atlas.org) of available chromatin immunoprecipitation sequencing (ChIP-Seq) data from the lymphoblastic cell line (LCL) GM12878 and the Hodgkin’s lymphoma (HL) cell line L1236 (not shown).

### LMP1 upregulates p62 expression via NF-κB and AP1 in EBV latency.

**FIG 2:**
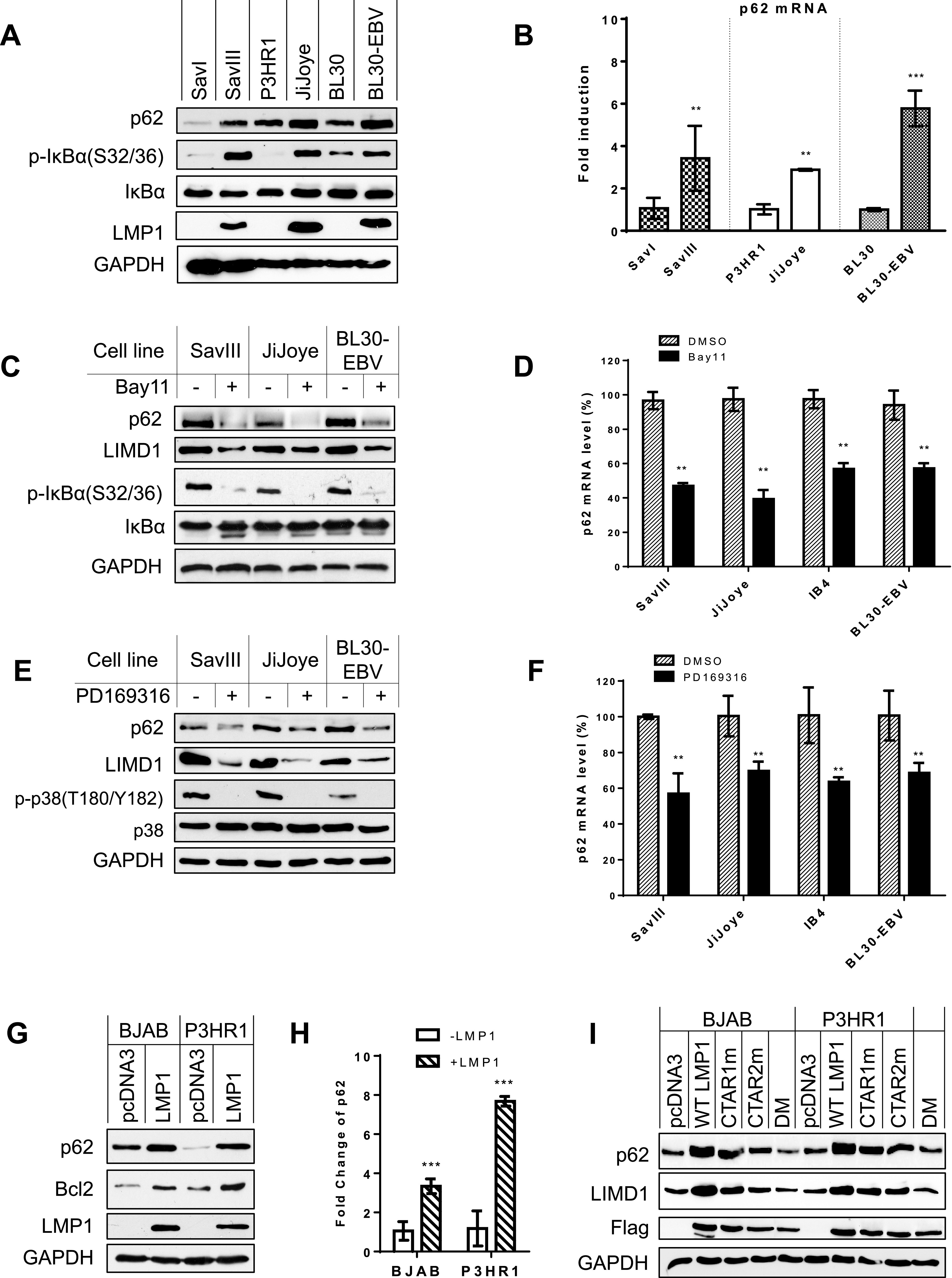
p62 is upregulated by NF-κB and AP1 downstream of LMP1 signaling. (A and B) p62 mRNA and protein expression levels are correlated with NF-κB activity in paired B cell lines. RNA was extracted from indicated different pairs of cell lines, and p62 mRNA expression level was evaluated by real-time qPCR. Consistent results were obtained from at least three independent repeats, and representative results from these consistent repeats are shown. The average mRNA levels of the triplicates in SavI, P3HR1, and CEM were set to 1. (C and D) Inhibition of NF-κB activity in virus-transformed cells downregulates p62 expression. NF-κB activity in type 3 latency cells was inhibited with the NF-κB-specific inhibitor Bay11-7085 at a concentration of 2.5 μM for 48 h. The average mRNA levels of the duplicates in dimethyl sulfoxide (DMSO)-treated cells were set to 100%. The p62 mRNA levels decreased by Bay11-7085 treatment are shown as percentages of those with corresponding DMSO controls. (E and F) Inhibition of p38 activity in virus-transformed cells downregulates p62 expression. PD169316 was applied at 5 nM for 48 h. (G and H) LMP1 induces p62 expression. BJAB and P3HR1 stable cell lines expressing Flag-LMP1 or controls were generated by transfecting with pLXCN/Flag-LMP1 expression and control plasmids, selected with 2 mg/ml G418 for 2 weeks, and then subjected to immunoblotting and qPCR analysis. The average mRNA levels of the triplicates in pcDNA3-transfected cells were set to 1. (I) Both LMP1 CTAR1 and -2 contribute to p62 expression. BJAB and P3HR1 cells were transfected with Flag-LMP1 or its point mutants. Cells were collected 48 h post-transfection, followed by immunoblotting with indicated antibodies. Statistical analysis was performed on results from three independent experiments. **, *P* < 0.01; ***, *P* < 0.001.

We then compared p62 expression levels in EBV type 1 and 3 latency programs in paired cell lines, which do not (type 1) or do (type 3) express high levels of LMP1. Results show that p62 mRNA and protein levels are higher in EBV type 3 latency compared with type 1 latency and correlate with LMP1 and NF-κB activity [p-IκBα(S32/36)]. Specifically, the p62 mRNA and protein levels are low in Sav I (type 1), P3HR1 (type 3, but LMP1 negative), and BL30 (EBV negative) but higher in the paired cell lines Sav III (type 3), JiJoye (type 3, LMP1 positive), and BL30-EBV (type 3) (Fig. 2A and B). P3HR1 was derived from JiJoye but has a very low level of LMP1, if any, due to the deletion of EBNA2, which is the master inducer of LMP1 expression.

Inhibition of the endogenous NF-κB activity by Bay11-7085 in type 3 latency significantly reduces p62 mRNA and protein levels (Fig. 2C and D). As a positive control, LIMD1, which is induced by LMP1/NF-κB as we showed recently (37), is also downregulated by NF-κB inhibition (Fig. 2C). Similar results were obtained with the p38-specific inhibitor PD169316 (Fig. 2E and F). We further found that p62 (Bcl-2 as a positive control) protein and mRNA levels are induced in BJAB and P3HR1 cell lines stably expressing LMP1 compared with those expressing a green fluorescent protein (GFP) control (Fig. 2G and H). Moreover, using the LMP1 point mutants (40) CTAR1m (LMP1-IID), which lacks TRAF-binding ability in CTAR1, CTAR2m (LMP1-PQAA), which lacks TRAF-binding ability in CTAR2, and DM (LMP1-IID/PQAA), which lacks TRAF-binding ability in both CTAR1 and -2, we confirmed that both CTARs contribute to p62 induction (Fig. 2I).

Note that it is not necessary that p62 mRNA and protein levels correlate closely in all paired cell lines, since we recently showed that p62 protein levels are also regulated by autophagy (30), and preliminary results further show that p62 transcription is regulated by NRF2 in response to oxidative stress, in these cells (not shown).

Taken together (Fig. 1 and 2), these results demonstrate that p62 is induced by NF-κB and AP1, which are activated downstream of EBV LMP1 signaling.

### LMP1 indirectly interacts with p62 via TRAF6.

**FIG 3:**
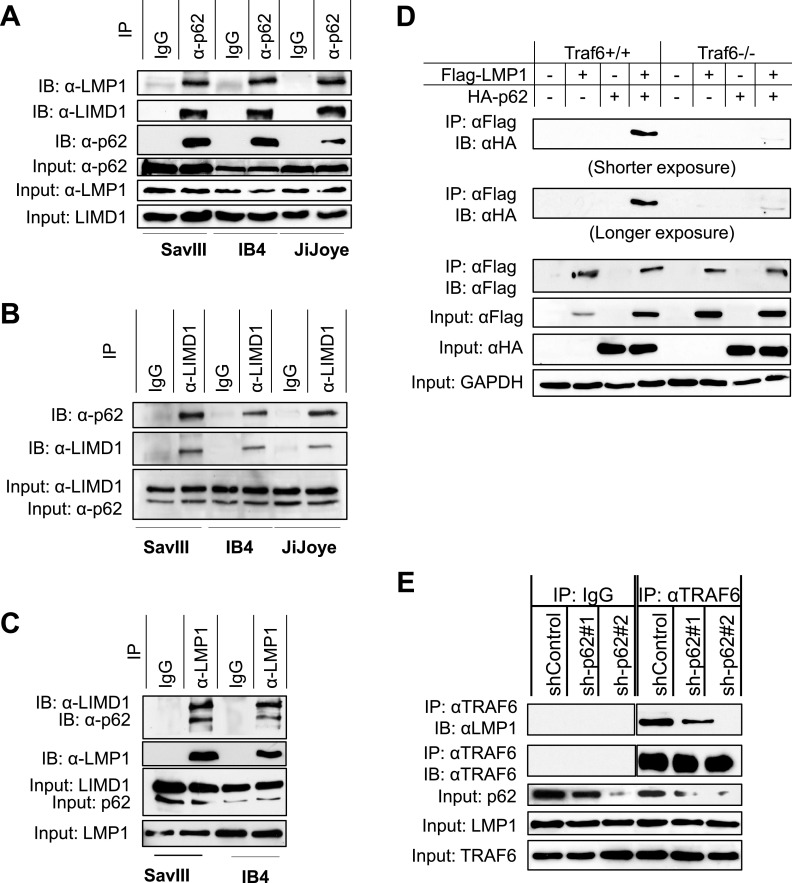
p62 interacts with TRAF6, LMP1 and LIMD1 in EBV latency. (A to C) Endogenous p62, TRAF6, LMP1, and LIMD1 interact with each other in EBV latency. EBV^+^ cells (type 3 latency) were subjected to IP and then IB with the indicated mouse antibodies. (D) TRAF6 mediates LMP1-p62 interaction. TRAF6^+/+^ and TRAF6^−/−^ MEFs were transfected with the indicated plasmids using Lipofectamine (Invitrogen). Cells were collected after 48 h for IP. (E) p62 deficiency impairs LMP1-TRAF6 interaction. IB4 cells expressing control or p62 shRNAs were induced with doxycycline (1 μg/ml) for 7 days and then subjected to IP with a rabbit TRAF6 antibody (Invitrogen), followed by IB with the indicated antibodies.

To examine the potential role of p62 in LMP1 signal transduction, we first sought to determine whether p62 interacts with LMP1 or LIMD1 in virus-transformed cells; LIMD1 was shown to interact with LMP1 in our recent publication (37). Immunoprecipitation (IP) results clearly show that endogenous p62, LMP1, and LIMD1 interact with each other in EBV latency (Fig. 3A to C).

**FIG 4:**
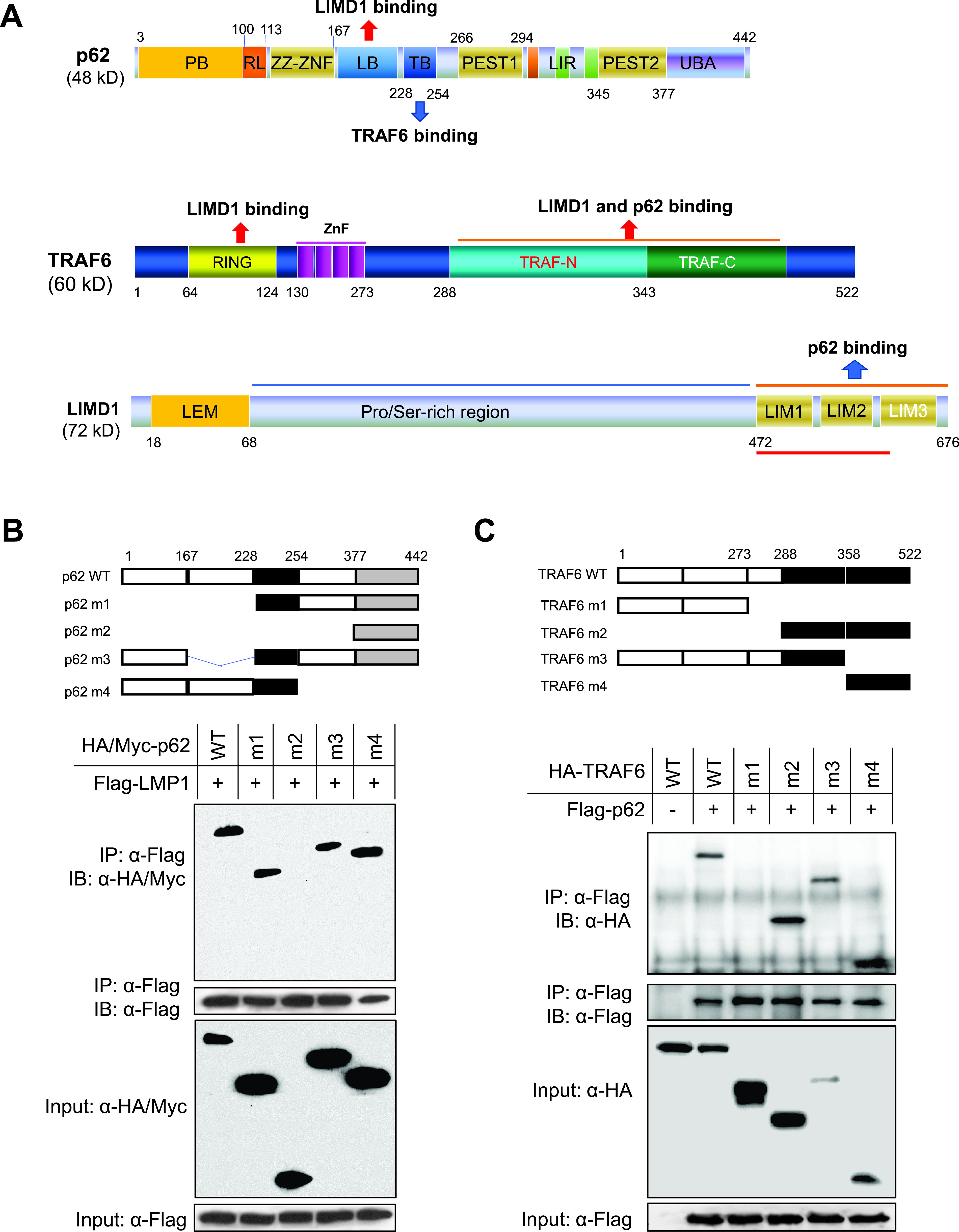
Mapping the regions on p62 and TRAF6 for their interaction. (A) Scheme of human p62, TRAF6, and LIMD1 proteins. (B) The TRAF6-binding domain of p62 interacts with LMP1. (C) The C-terminal TRAF domains of TRAF6 are required for its interaction with p62. 293T cells in 60-mm dishes were transfected with indicated plasmids (HA-tagged WT p62 and m1, m2, and m4 and Myc-tagged p62 m3) using Fugene HD reagent (Promega) and collected 48 h later for IP and then IB with indicated antibodies.

As a signal-transducing adaptor, p62 has been documented to directly interact with TRAF6, leading to NF-κB activation in diverse biological contexts (26, 41, 42). LMP1 is well known to interact with TRAF6 and other TRAF family members (8, 43). Thus, we next aimed to examine the requirement of TRAF6 for LMP1-p62 interaction. To this end, we transfected TRAF6^+/+^ and TRAF6^−/−^ mouse embryo fibroblasts (MEFs) with Flag-LMP1 and hemagglutinin (HA)-tagged p62 and collected the cell lysates after 48 h, followed by IP to evaluate LMP1-p62 interaction. We detected LMP1-p62 interaction in TRAF6^+/+^ MEFs but not in TRAF6^−/−^ MEFs, indicating that TRAF6 mediates LMP1-p62 interaction (Fig. 3D). To confirm the role of TRAF6 in mediating LMP1-p62 interaction in EBV latency, we performed IP for endogenous LMP1-TRAF6 interaction in IB4 cells stably expressing p62-specific shRNAs (as usual, to reduce potential “off-target” effects on interpreting the outcomes, we chose two p62 shRNAs) (30). Results show that p62 deficiency substantially impairs LMP1-TRAF6 interaction after 7 days with doxycycline induction of p62 shRNA expression (Fig. 3E). This conclusion was confirmed with the use of a panel of p62 deletion mutants, which show that the TRAF6-binding motif on p62 is required for p62 interaction with LMP1 (see Fig. 4B).

Together, these results demonstrate that p62 is a component of the LMP1 signalosome through its physical interaction with TRAF6.

### Mapping the regions on p62 and TRAF6 for p62-TRAF6 and p62-LMP1 interactions.

Since TRAF6, LIMD1, and p62 have been shown to interact with each other, we next aimed to determine whether TRAF6, LIMD1, and p62 are in the same complex for LMP1 signal transduction.

To this end, we first used a panel of deletion mutants of p62 and TRAF6 to map the domains responsible for their interaction with each other and also with LMP1 (Fig. 4A). Results show that the TRAF6-binding domain (TB) of p62 is responsible for its interaction with LMP1 (Fig. 4B), further supporting the claim that TRAF6 mediates the interaction between p62 and LMP1.

Like p62, LIMD1 can also directly interact with TRAF6, and we have shown that LIMD1 also interacts with LMP1, as evaluated by IP (37). Thus, TRAF6 mediates both p62-LMP1 and LIMD1-LMP1 interactions. It was necessary next to determine if p62 and LIMD1 compete for TRAF6 binding by targeting the same region. It is known that the TRAF domains, as well as the N-terminal RING, of TRAF6, can interact with LIMD1 (44). The domain responsible for p62 interaction has not yet been mapped. We performed IP for this purpose, and results show that both TRAF-N and TRAF-C of TRAF6 can interact with p62 (Fig. 4C).

### p62 promotes LMP1-stimulated TRAF6 ubiquitination.

**FIG 5:**
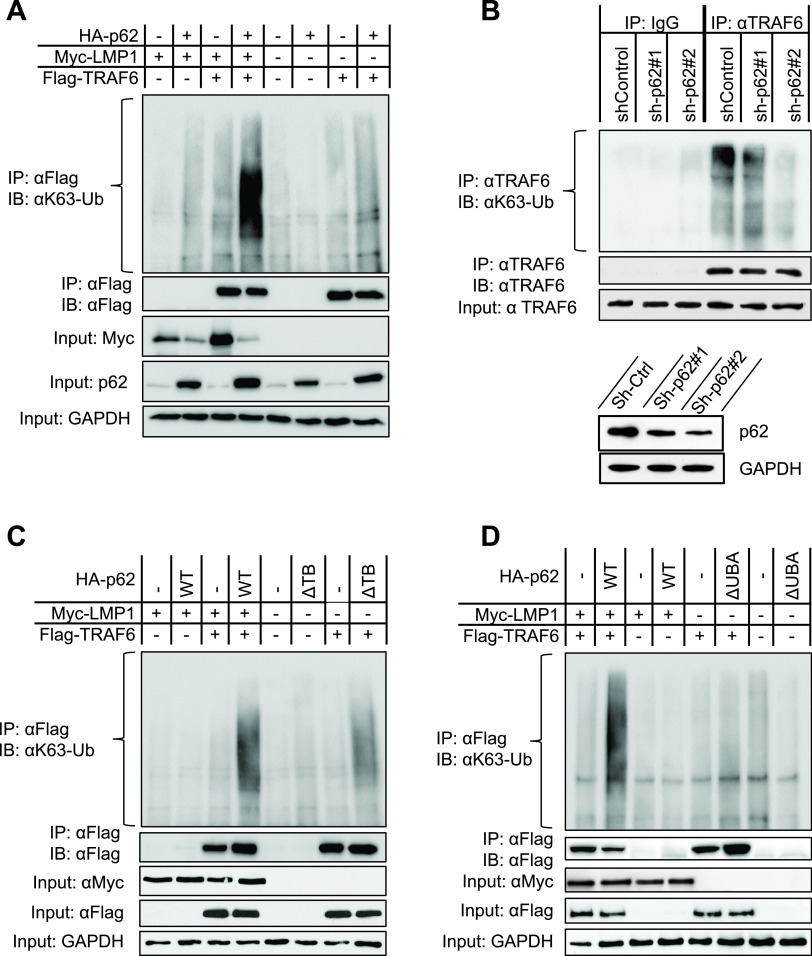
p62 promotes LMP1-mediated TRAF6 ubiquitination. (A) p62 enhances LMP1-stimulated TRAF6 ubiquitination. (B) p62 deficiency impairs endogenous TRAF6 ubiquitination. IB4 cell lines stably expressing p62 shRNA#1 and shRNA#2 (or control shRNA) were induced by 1 μg/ml doxycycline for 48 h for shRNA expression, and cell lysates were then subjected to denaturing IP with a TRAF6 antibody (Invitrogen), followed by IB with the indicated antibodies. (C and D) TRAF6- and the ubiquitin-binding domains (UBA) of p62 are required for its ability to promote LMP1-mediated TRAF6 ubiquitination. For panels A, C, and D, 293T cells in 60-mm dishes were transfected with indicated plasmids. Forty-eight hours later, cells were collected, and denaturing IP was performed with anti-Flag, followed by IB with the indicated antibodies. Consistent results were obtained from at least three independent repeats, and representative results are shown.

As a ubiquitin sensor, p62 has been reported to promote TRAF6 oligomerization, facilitating TRAF6 self-ubiquitination (41, 42, 45). To test whether p62 facilitates LMP1-mediated TRAF6 ubiquitination, we first transfected different combinations of tagged LMP1, p62, TRAF6, and ubiquitin (Ub) plasmids into 293T cells, followed by denaturing IP. As shown in Fig. 5A, LMP1 stimulates TRAF6 K63-linked polyubiquitination, which is further enhanced in the presence of p62. Of note, p62 cotransfection results in a consistent decrease of the LMP1 protein level, which may be caused by p62-mediated autophagic degradation, as shown in our recent publication (30). We then assessed endogenous TRAF6 ubiquitination in IB4 cells with shRNA-mediated p62 knockdown (KD), and the results clearly show that p62 deficiency significantly reduces TRAF6 K63-linked polyubiquitination (Fig. 5B). These results indicate that p62 potentiates LMP1-stimulated TRAF6 polyubiquitination.

Binding of the UBA of p62 to K63 ubiquitin chains is not only required for p62-mediated autophagic degradation, but also for p62-mediated NF-κB activation (25). The UBA of p62 may facilitate the recruitment of ubiquitin molecules to TRAF6. We next aimed to evaluate whether the TRAF6-binding domain (TB) and ubiquitin-binding domain (UBA) of p62 are required to promote LMP1-stimulated TRAF6 ubiquitination. We transfected wild-type (WT) HA-p62, HA-p62(ΔTB) or HA-p62(ΔUBA), along with Myc-LMP1, Flag-TRAF6, and HA-Ub plasmids into 293T cells, and the cell lysates were collected after 48 h for denaturing IP with anti-Flag. As shown in Fig. 5C and D, the deletion of either the TB or UBA domain of p62 consistently reduces TRAF6 ubiquitination stimulated by LMP1, indicating that both domains are required for LMP1-stimulated TRAF6 ubiquitination.

Taken together (Fig. 3 and 5), these findings indicate that p62 participates in LMP1 signal transduction through its interaction with TRAF6, promoting TRAF6 ubiquitination and activation.

### p62 mediates LMP1 signaling to activation of NF-κB, AP1, and Akt.

**FIG 6:**
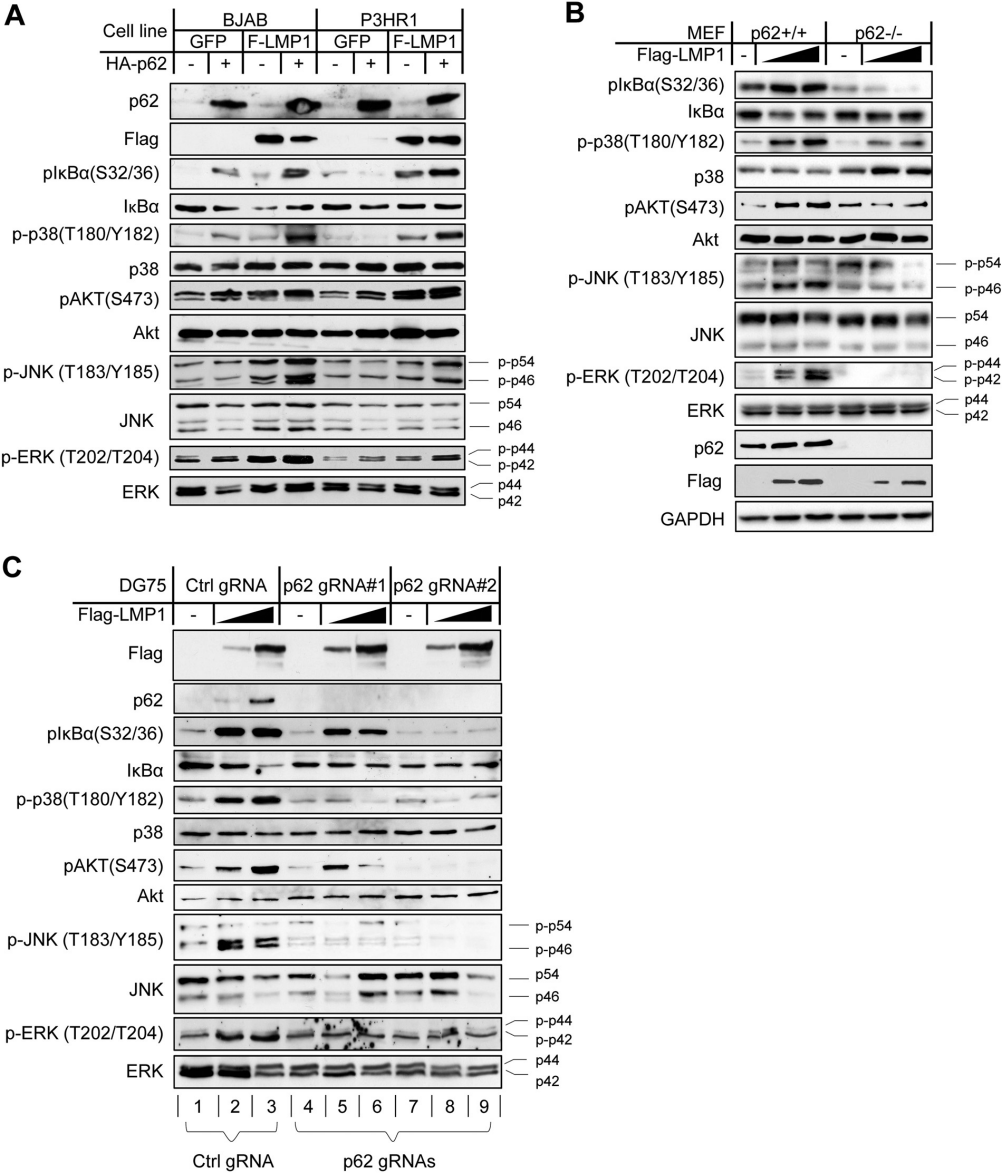
p62 is required for LMP1 activation of NF-κB, mitogen-activated protein kinases (MAPKs), and Akt. (A) Exogenic expression of p62 stimulates activation of NF-κB, MAPKs, and Akt in the presence of LMP1. BJAB and P3HR1 cells stably expressing Flag-LMP1 (F-LMP1) or GFP control were transfected with HA-p62. Site-specific phosphorylation of IκBα, MAPKs, and Akt representative of their activation was evaluated 48 h later. (B) LMP1 stimulates activation of NF-κB, MAPKs, and Akt in p62^+/+^ MEFs but not in p62^−/−^ MEFs. MEFs were transfected with Lipofectamine, followed by IB with the indicated antibodies after 48 h. (C) CRISPR-mediated p62 KO abrogates LMP1 activation of NF-κB, MAPKs, and Akt. DG75 cells were transfected with p62-specific sgRNAs or control (Luc) cloned in pLenti-CRISPRv2 eSpCas9, followed by selection with puromycin (0.5 μg/ml) for 2 weeks. Selected cells were then transfected with the indicated plasmids, and IB was conducted with the indicated antibodies after 48 h. Consistent results obtained from three independent repeats are shown.

TRAF6 is required for LMP1 signaling to NF-κB and AP1 activation, and was also shown to mediate LMP1 activation of Akt (46). Since we show that p62 interacts with TRAF6, we next aimed to determine the requirement of p62 in TRAF6-mediated LMP1 signaling to activation of NF-κB, AP1, and Akt. To this end, we first transfected HA-p62 plasmids into BJAB or P3HR1 cells stably expressing Flag-LMP1 or GFP control. Results show that transient expression of HA-p62 markedly upregulates endogenous levels of phosphorylated forms of IκBα, p38, Akt, JNK (especially the p46 subunit [the lower band]), and ERK (Fig. 6A).

We then tested the requirement of p62 for LMP1 activation of these downstream effectors in p62^−/−^ MEFs. As shown in Fig. 6B, Flag-LMP1 transfection promotes phosphorylation of IκBα, p38, Akt, the p46 subunit of JNK and ERK in p62^+/+^ MEFs but not in p62^−/−^ MEFs, indicating that p62 is indispensable for LMP1 signal transduction.

To test in more biologically relevant contexts, we employed a CRISPR/Cas9-mediated gene-editing technique that can remove a designated gene in the target cell to completely deplete its expression in response to any stimulus. We first tried to use CRISPR/Cas9 to knock out the p62-encoding gene with the construct pLenti-CRISPR/eSpCas9 expressing p62-specific small guide RNA (sgRNA) or control sgRNA in EBV latency 3 (SavIII and JiJoye cells). As a consequence of the CRISPR/Cas9-mediated knockout (KO), Burkitt’s lymphoma (BL) cell lines SavIII and JiJoye without the p62-encoding gene failed to survive (data not shown), making further analysis impossible, although we may find some EBV-transformed lymphoblastic cell lines (LCLs) that can tolerate the removal of the p62-encoding gene. Indeed, this outcome implies that p62 is crucial for the survival of EBV-transformed cells. Thus, we switched to EBV-negative BL cell lines. To this end, we transfected DG75 cells with these p62 or control sgRNA plasmids, followed by antibiotic selection (puromycin) to deplete untransfected cells. Again, we chose two p62-specific sgRNA sequences to reduce potential off-target effects on interpreting the outcomes. Then, we transfected the antibiotics-selected stable polyclonal cells with Flag-LMP1, followed by immunoblotting analysis after 2 days. Results show that transient expression of LMP1 stimulates phosphorylation of IκBα, p38, Akt, JNK, and ERK in control sgRNA-expressing cells (Fig. 6C, lanes 1 versus 2 and 3) but not in p62 sgRNA-expressing cells (Fig. 6C, lanes 4 versus 5 and 6 and lanes 7 versus 8 and 9).

To further validate this conclusion in EBV latency that p62 is required for TRAF6-mediated LMP1 signaling, we employed shRNA-mediated knockdown (KD) to downregulate endogenous p62 transcripts in EBV-transformed cells, followed by immunoblotting for phosphorylation of the above-mentioned LMP1 downstream effectors. Surprisingly, shRNA-mediated KD of p62 transcripts (approximately 60 to 70% KD efficiency in p62 shRNA stable cells) did not have consistent or remarkable effects on phosphorylation of LMP1 effectors, as evaluated by immunoblotting (data not shown), despite many attempts. To interpret the unexpected results, we reason that the changes in the phosphorylation of these effectors in response to shRNA-mediated KD of p62 transcripts are not detectable by immunoblotting.

**FIG 7:**
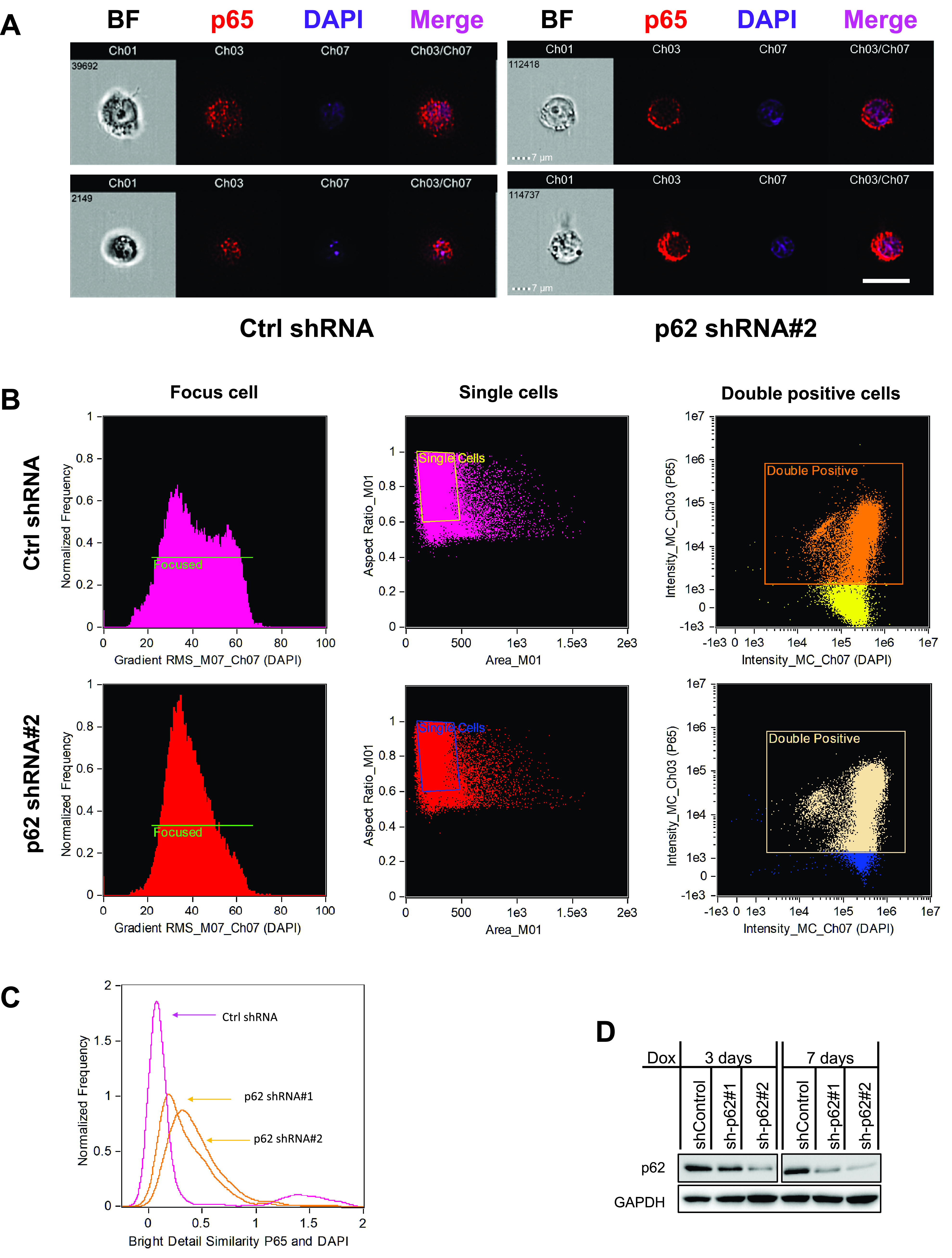
p62 is required for p65 nuclear translocation in EBV-infected cells. IB4 cells stably expressing p62 shRNAs or control shRNA were treated with 1 μg/ml doxycycline for 3 days and subjected to immunostaining with mouse p65 antibody (2A12A7; Invitrogen), followed by anti-mouse immunoglobulin Alexa 555. Cells were then subjected to Amnis imaging flow analysis. Images were acquired and processed in IDEA software. The images of a single cell from different channels were aligned automatically in a row. Representative rows of images are shown. (A) Single-cell images were acquired, and a representative gallery of single cells from control and p62 shRNA#2 is shown. Bar = 10 μm. (B and C) Cell population analysis for p65 subcellular localization using IDEA software, as described in Materials and Methods. (D) Immunoblotting analysis shows p62 KD efficiency at day 3 and day 7 with doxycycline induction.

To overcome this limitation, we assessed p65 subcellular localization by Amnis imaging flow cytometry, which can quantitate protein subcellular localization at both the cell population and single-cell levels simultaneously. To this end, IB4 cells stably expressing p62 shRNAs (or control shRNA) were subjected to Amnis analysis, followed by data processing in the IDEA program. As shown in Fig. 7A, at the single-cell level, the subcellular localization of p65 has no apparent difference in cells expressing control shRNA compared to p62 shRNA. In each sample, various p65 subcellular distributions can be detected in different cells. However, quantitative analysis for cell populations shows that p62 KD results in a remarkable decrease of the cell population (normalized frequency) with p65 in the nucleus, which is expressed by the median similarity score (SS) (47) (Fig. 7B and C). These results clearly show that p62 is required for p65 nuclear translocation indicative of NF-κB activation.

Altogether, our results indicate that p62 is required for TRAF6 to mediate LMP1 signal transduction, through its interaction with TRAF6.

### p62 is required for LMP1 regulation of target gene expression.

**FIG 8:**
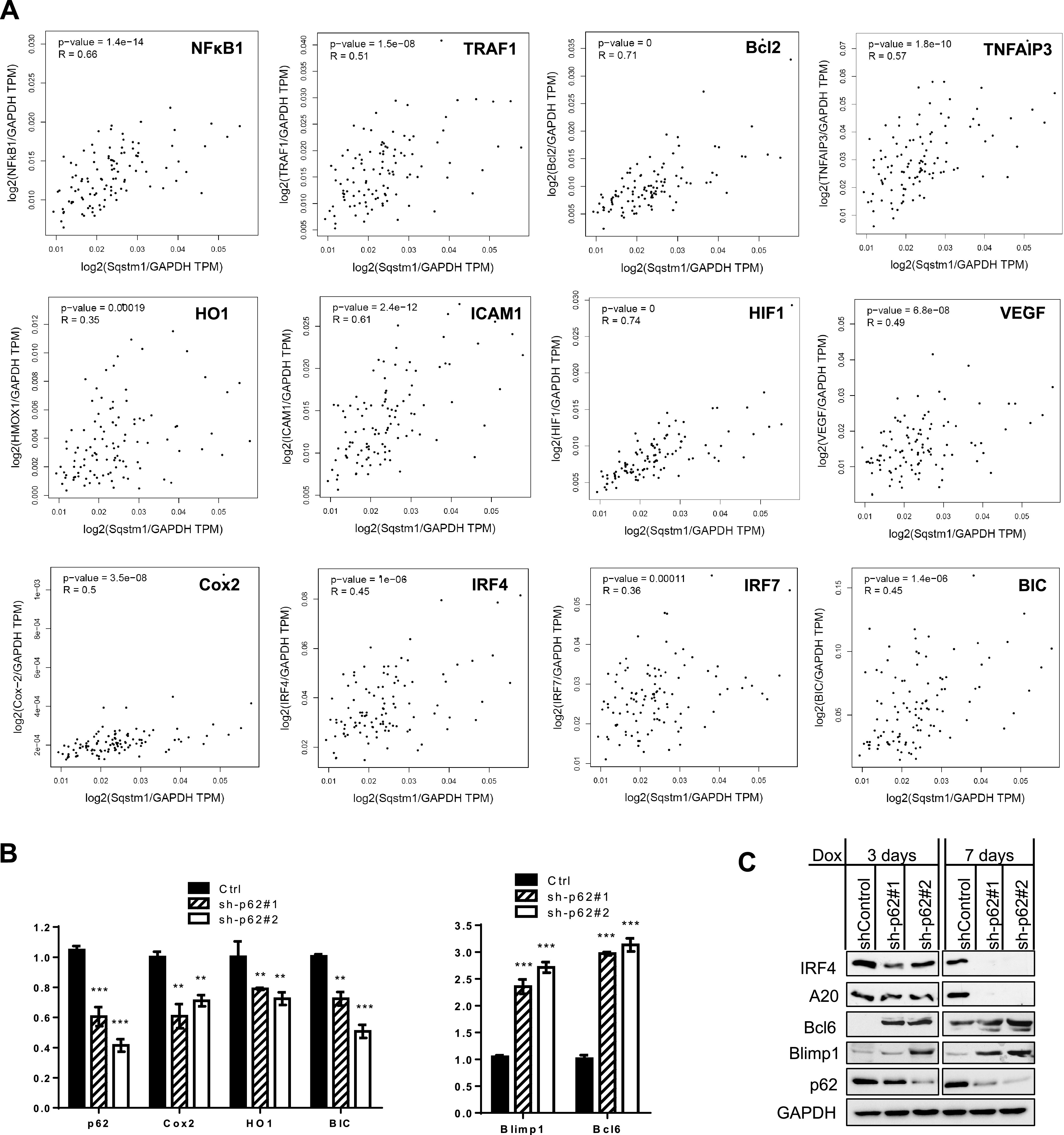
p62 is required for LMP1 regulation of target gene transcription. (A) Pearson correlation analysis was carried out using the algorithm GEPIA v2.0 for p62 and selected LMP1 target genes. (B) shRNA-mediated p62 KD in IB4 cells deregulates selected LMP1 target genes at the mRNA level. Cells were collected 3 days after 1 μg/ml doxycycline induction, and then qPCR was performed. Consistent results were obtained from at least three independent repeats, and representative results from these consistent repeats are shown. (C) shRNA-mediated p62 KD in IB4 cells deregulates selected LMP1 target genes at the protein level.

We next sought to evaluate the requirement of p62 for LMP1 target gene expression. First, we evaluated the correlation of mRNA expression between p62 and LMP1 target genes, using the algorithm Gene Expression Profiling Interactive Analysis (GEPIA v2) (48), which includes the GTEx portal with the data set derived from 174 EBV-transformed LCLs. Results show that p62/Sqstm1 transcript significantly (*P* < 0.05; *R* > 0.23) correlates with those of a pool of selected LMP1 targets, such as NFκB1/p50, TRAF1, BclII, A20/TNFAIP3, HMOX1/HO1, ICAM1, VEGF, Cox2, IRF4, IRF7, and miR-155HG/BIC (Fig. 8A) but not with those of other well-characterized LMP1 target genes, such as LIMD1 (*P* = 0.27, *R* = 0.11), Blimp1/PRDM1 (*P* = 0.08, *R* = 0.17), and VIM (*P* = 0.39, R = −0.084) (data not shown). For LIMD1, our immunoblotting results show that it correlates with p62 very well at the protein and mRNA levels in EBV latency, and both are induced by LMP1/NF-κB and LMP1/AP1 (Fig. 2C to F). These controversial results indicate that the data set in the GTEx portal, which was obtained from high-throughput RNA sequencing, has limitations on some genes, and further experimental validation is needed.

We further performed real-time quantitative PCR (qPCR) to validate the requirement of p62 for the regulation of selected LMP1 target genes, using gain- and loss-of-function approaches. As mentioned above, CRISPR/Cas9-mediated p62 knockout (KO) in EBV-positive BL cells (type 3 latency) causes rapid cell death. We thus conducted this experiment in IB4 cells with shRNA-mediated p62 KD, and results show that shRNA-mediated KD of p62 remarkably deregulates LMP1 target genes, including the downregulation of the oxidative stress-related genes COX2 and HO1 and miR-155HG/BIC and the upregulation of Blimp1 and Bcl6 (Fig. 8B).

We also evaluated selected LMP1 targets at the protein level, and results show that p62 deficiency deregulates their protein levels (Fig. 8C). Some LMP1 target genes (for example, Bcl6), which show poor expression at the mRNA level probably due to the quality of the corresponding probes in the transcriptome sequencing (RNA-Seq), however, express high protein levels and are remarkably deregulated in p62-deficient cells compared to cells expressing shRNA control.

### p62 is required for LMP1-mediated LCL outgrowth.

**FIG 9:**
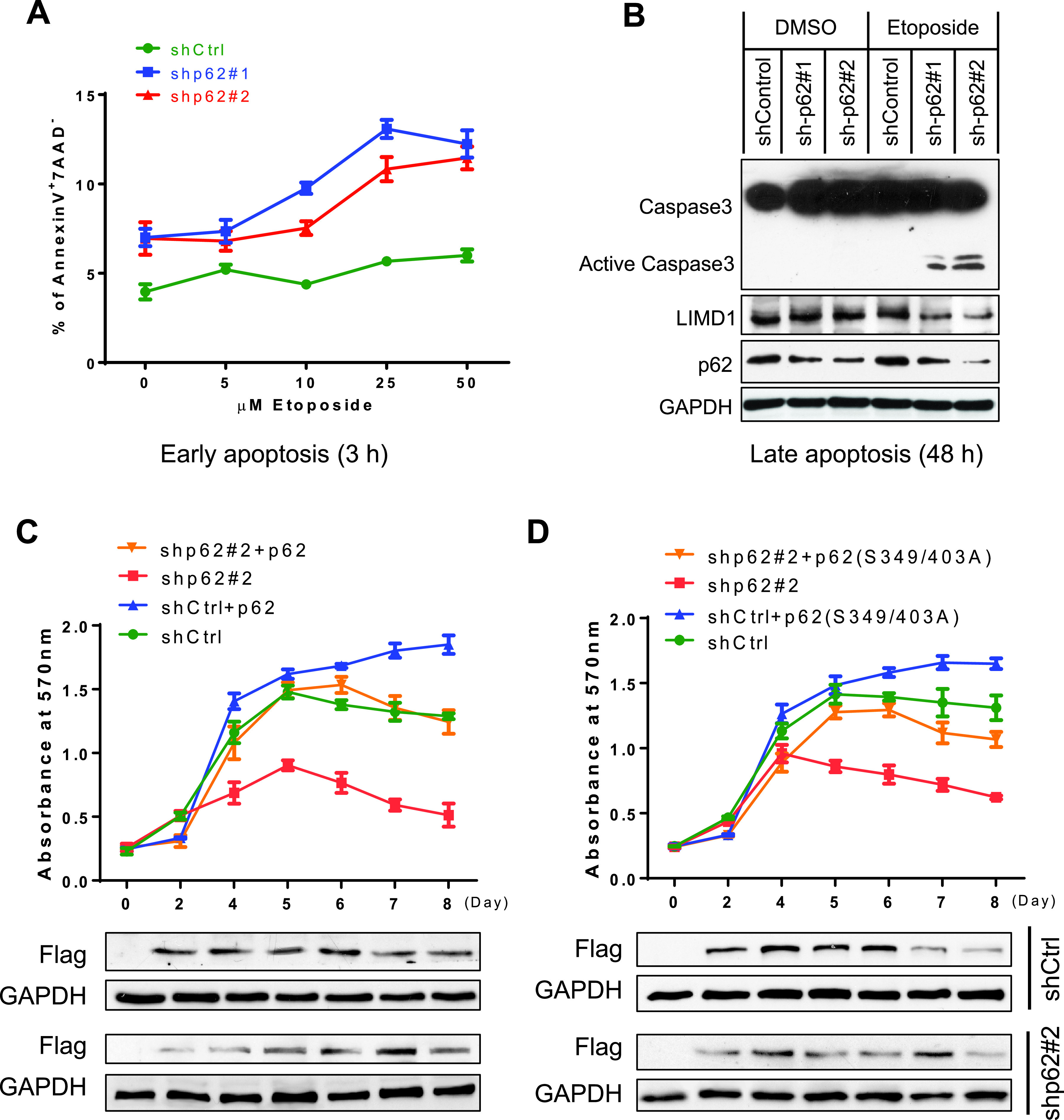
p62 contributes to LCL outgrowth and proliferation. (A and B) shRNA-mediated p62 deficiency promotes etoposide-induced cell death. IB4 cell lines stably expressing p62 shRNA#1 and shRNA#2 (or control shRNA) were induced by 1 μg/ml doxycycline for 4 days, followed by treatment with etoposide at different concentrations and for different times (A) and with 5 μM etoposide (B). Doxycycline was replaced every 4 days to maintain shRNA expression. Early and late apoptosis was evaluated, and representative results are shown. (C and D) p62 deficiency reduces cell proliferation, which can be rescued in part by wild-type p62 or the phosphorylation-deficient mutant p62(S349/403A). IB4 cell lines stably expressing p62 shRNA#2 targeting the 3′ UTR (or control shRNA) were transfected with Flag-p62 or Flag-p62(S349/403A) using a Nucleofector B cell kit (Lonza); 1 μg/ml doxycycline was added to induce shRNA expression at the time of transfection. Cell proliferation was measured for up to 8 days with doxycycline.

LMP1 is essential for the outgrowth of EBV-transformed B cells *in vitro* (2, 49), and its deficiency can result in DNA damage in EBV latency, leading to cell death (50). Since KO of the p62-encoding gene SQSTM1 in EBV-transformed BL cells results in rapid cell death, we thus sought to evaluate functional outcomes in IB4 cells with shRNA-mediated KD of p62 transcripts. Flow cytometry results show that, although shRNA-mediated p62 deficiency in IB4 cells alone did not cause cell death, it aggravated apoptosis induced by etoposide and elevated caspase 3 activity (Fig. 9A and B).

p62 partial deficiency also downregulates cell proliferation. Importantly, this downregulation can be rescued in part by wild-type p62, or by p62(S349/403A), a phosphorylation-deficient mutant that retains the ability of p62 to interact with TRAF6 but has lost the functions in autophagy and antioxidative stress (Fig. 9C and D) (51). This rescue strategy is feasible in that the p62 shRNA#2 we chose targets p62 mRNA 3′-UTR, which is approximately 1.5 kb in length and has multiple potential shRNA-targeting sites.

Together, these results indicate that p62 is critical for LMP1-mediated LCL outgrowth and proliferation and that LMP1 protects EBV-positive cells from the proapoptotic activity of p62 deficiency.

## DISCUSSION

In this study, we present solid evidence showing that the ubiquitin sensor and signal-transducing adaptor p62 is upregulated by NF-κB and AP1, which are specifically activated downstream of the EBV LMP1 signaling pathway in EBV-infected cells. Moreover, p62 interacts with TRAF6 and stimulates its ubiquitination, promoting LMP1 signal transduction to the activation of NF-κB, AP1, and Akt in a positive regulatory loop. These findings, by taking p62 as a novel player in these settings, provide profound mechanistic insights into signal transduction mediated by LMP1.

**FIG 10:**
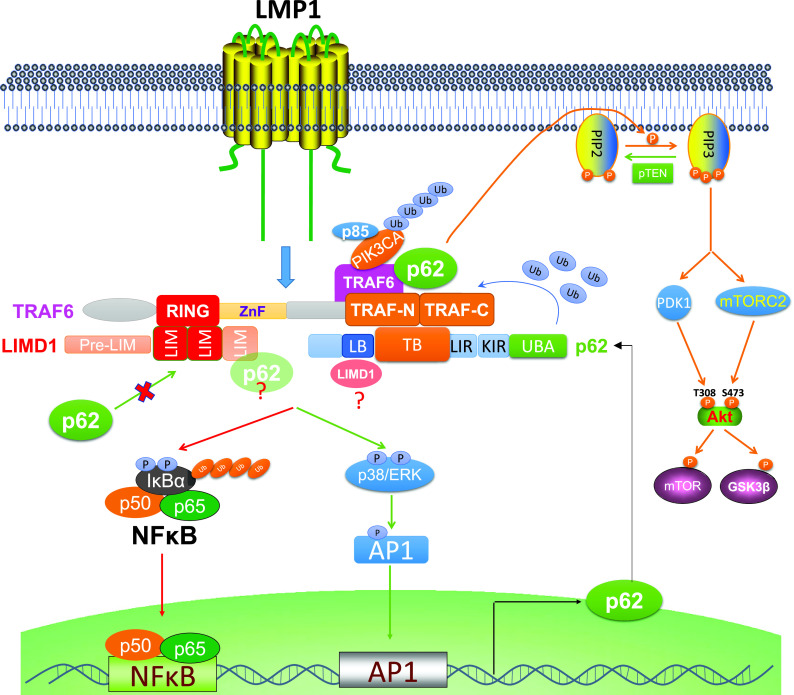
Diagram showing the interplay between p62 and LMP1 signal transduction. LMP1 induces p62 expression via NF-κB and AP1 axes. In return, p62 participates in LMP1 signal transduction through its interaction with TRAF6, promoting TRAF6 ubiquitination. TRAF6 C-terminal TRAF-N/C domains bind to the TB domain on p62, and the LB domain of p62 can further bind to LIMD1. In the meantime, TRAF6 N-terminal RING binds to LIMD1 C-terminal LIM1/2 domains, excluding p62 in that p62 also binds to LIMD1 LIM domains (an unlikely possibility is that p62 can further binds to LIM3 of LIMD1).

LMP1 directly interacts with TRAF6 (2, 8, 52). Previously, we identified the adaptor protein LIMD1 as a novel component of the LMP1 signalosome through its interaction with TRAF6 (37). p62 has well-defined interacting motifs for both TRAF6 and LIMD1 and is known to interact with LIMD1 in the LIMD1-p62-TRAF6-PKCζ multiprotein complex during osteoclast development (44, 53). It is also known that LIMD1 can bind to both N-terminal RING and C-terminal TRAF domains (44), but p62 binds only to C-terminal TRAF domains (41) (Fig. 5A). Therefore, there are two possibilities for TRAF6 interaction with LIMD1 and p62 downstream of LMP1: (i) TRAF6 interacts only with LIMD1, with both N-terminal RING and C-terminal TRAF domains of TRAF6 being occupied by LIMD1, excluding p62 binding, or (ii) TRAF6 simultaneously interacts with both LIMD1 and p62. In this case, the RING is occupied by LIMD1, but the TRAF domains are occupied by p62. Our functional analyses indicate that both LIMD1 and p62 are required for TRAF6-mediated LMP1 signal transduction, implying that these three proteins are in the same signalosome downstream of LMP1 signaling, with the TRAF6 N-terminal RING domain binding to LIMD1 and TRAF6 C-terminal TRAF domains binding to p62 (Fig. 10).

There is a possibility that p62 in the TRAF6-p62-LIMD1 complex can further interact with a second LIMD1 in that the TRAF6- and LIMD1-binding domains on p62 are distinct (Fig. 5A and 10). Moreover, the C-terminal LIM domains are responsible for binding to both TRAF6 and p62 (Fig. 5A). A higher-resolution map shows that the LIM1/2 domains are responsible for TRAF6 interaction (44). Thus, there is a minor possibility that LIMD1 in the TRAF6-p62-LIMD1 complex can further bind to p62 through the LIM3 domain (Fig. 10). Nevertheless, both functional and mapping studies by us and others support the claim that TRAF6, LIMD1, and p62 exist in the same complex for LMP1 signal transduction.

It has been documented that p62 specifically interacts with TRAF6, but not with TRAF5 or TRAF2, to facilitate TRAF6 K63-linked ubiquitination (41, 42, 45). However, p62 may interact with TRAF1 to contribute to LMP1 signal transduction, since TRAF1 was identified as an interacting partner for p62 in a high-throughput screen (32). As another TRAF family member, TRAF1 is involved in LMP1 signal transduction by interacting with LMP1 C-terminal activating region 1 (CTAR1). In fact, in TRAF6^−/−^ cells, we could detect a weak signal after longer exposure, indicating the LMP1-p62 interaction in IP assays (Fig. 3D) and suggesting that TRAF1 may also mediate LMP1-p62 interaction. Further investigation is needed to validate the p62-TRAF1 interaction and its possible role in LMP1 signal transduction.

p62 has been previously reported to promote NF-κB activation in diverse contexts (24, 25, 54–58), including CD40 signaling (58), which mimics LMP1 signaling, in B cells. However, our loss-of-function assays using shRNA-mediated p62 KD failed to show a consistent downregulation of IκBα phosphorylation by p62 KD, as evaluated by immunoblotting. In agreement with these results obtained via an RNA interference strategy, a recent report also showed that ectopic expression of p62 mediates HTLV-1 Tax activation of NF-κB in multiple cell types, but immunoblotting results failed to show that small interfering RNA (siRNA)-mediated p62 deficiency in HTLV-1-transformed cells alters endogenous IKKα/β activity stimulated by HTLV-1 Tax (33), which resembles LMP1 in signal transduction in many respects (1). The failure to detect NF-κB activity deregulated by p62 shRNAs in virus-transformed cells may be attributable to the potential limitations of these assays.

To overcome these potential limitations in this study, we undertook three other strategies: (i) using Amnis flow cytometry to evaluate p65 subcellular localization instead of immunoblotting for analysis of the deregulation of the phosphorylation of NF-κB and p38, (ii) using CRISPR-mediated gene KO to replace shRNA-mediated mRNA KD, and (iii) evaluating the deregulation of different LMP1 target genes. These alternative strategies generated solid evidence to support the claim that p62 mediates LMP1 activation of NF-κB, AP1, and Akt, all of which require TRAF6. In fact, we also obtained similar results with HTLV-1-infected T cells using these alternative strategies (data not shown).

p62 has two predominant functions: as a signaling adaptor and as a ubiquitin sensor (25). Our current study demonstrates that it functions as a signaling adaptor that mediates TRAF6-mediated LMP1 signal transduction. As a ubiquitin sensor, p62 mediates selective autophagy that can selectively target ubiquitinated proteins for autophagic degradation, such as RIG-I (59, 60), cGAS (61), STING (62), and p62 itself, in the innate immune response. Recently, we showed that p62-mediated selective autophagy is constitutively induced in response to oxidative stress in EBV/HTLV-1 latency (30). In fact, the ubiquitin sensor function of p62 is also required for TRAF6-mediated NF-κB activation (Fig. 4) (24, 25). Thus, both roles of p62 are invoked by EBV/HTLV-1 latent infection.

Not only is p62 induced by LMP1 in EBV latency, but our recent publication and other preliminary data show that it is also induced by the master antioxidant transcription factor NRF2 under oxidative stress (30). In support of its role in antioxidant response in EBV latency, p62 also correlates or reversely correlates with many genes involved in oxidant stress at the transcriptional level in our algorithm analysis, such as Keap1, NRF2, SOD, and CAT (data not shown). In this regard, p62 likely plays a role in antioxidant stress in both EBV lytic/primary and latent infections, given that oxidative stress is a common feature of these events (63–66). Further pursuits on this topic are our next research priority.

In summary, this study identified p62 as a novel player in LMP1 signal transduction and oncogenic functions. The proposed mechanism may have broader significance in virus-mediated oncogenesis in that it is also shared by HTLV-1 Tax (33) and potentially by other viral oncogenic pathways. Further studies are needed to elaborate on the molecular mechanisms underlying the interaction of p62 with EBV/HTLV-1 infection, including its role in linking autophagy, DNA damage response, immune response, and cell death, which represent the complicated mechanisms responsible for viral latency and oncogenesis. These long-term pursuits may open up unique opportunities to target these mechanisms for therapeutic interventions.

## MATERIALS AND METHODS

### Cell lines.

SavI, SavIII, P3HR1, and JiJoye are human B cell lines derived from EBV-positive Burkitt’s lymphoma (BL) patients. P3HR1 was derived from JiJoye but does not express LMP1 because it lacks the entire EBNA2 open reading frame (ORF) in the viral genome (67). BL30 is an EBV-negative BL line, and BL30-EBV was derived from BL30 infected *in vitro* with EBV strain B95.8 (68). BJAB is an EBV-negative non-BL line. The lymphoblastic cell line (LCL) IB4 was derived from umbilical cord B lymphocytes latently transformed with EBV *in vitro*. B cell lines were cultured with RPMI 1640 medium plus 10% fetal bovine serum (FBS), and antibiotics. 293, 293T, and mouse embryo fibroblasts (MEFs) were cultured with Dulbecco’s modified Eagle medium (DMEM) plus 10% FBS and antibiotics. All cell culture supplies were purchased from Life Technologies.

### Antibodies and reagents.

p62 (D-3), LIMD1 (H-4), and caspase-3 (E-8) mouse monoclonal antibodies were from Santa Cruz. Mouse LIMD1(3F2C6), rabbit LIMD1, and rabbit LIMD1(N1-50) antibodies were purchased from Millipore or Sigma for different purposes. p65 (clone 2A12A7) for Amnis analysis was from Invitrogen. LMP1 (CS1-4) and TRAF6 (1H4L2) were from Dako and ABfinity, respectively. Phospho-specific antibodies and corresponding plain antibodies, K63-Ub, and horseradish peroxidase (HRP)-coupled secondary antibodies were from Cell Signaling Technologies. Mouse HA (clone HA-7), rabbit HA, and Flag (clone M2) antibodies were from Sigma. All other antibodies are from Invitrogen or eBioscience.

The human p62(−1781/+46)-Luc2 construct was provided by Terje Johansen (27, 34). HA-p62 and deletion mutants (m1, m2, and m4) were from Yu-Ying He, Myc-p62(m4) was from Ying Zhao (69, 70), and Flag-tagged p62 point mutants were from Ronggui Hu (51). TRAF6 and deletion mutants were from Ying Zhu (71). Additional p62 mutants were generated by site-directed mutagenesis and verified by sequencing.

Chemical inhibitors, including BAY11-7085, PD169316, and etoposide, were purchased from Sigma and EMD Millipore.

### CRISPR/Cas9- and shRNA-mediated targeting.

The p62-specific shRNA targeting sites were shp62#1 (5′-TCTCTTTAATGTAGATTCG; cDNA) and shp62#2 (5′-TGCCTAATGGCTTTCACTTTC; 3′ untranslated region [3′-UTR]). p62 shRNAs were cloned in the pTRIPz/puro doxycycline-inducible lentiviral vector. p62-specific CRISPR/Cas9 plasmids were generated by GenScript by cloning p62 sgRNAs into pLenti-CRISPRv2 eSpCas9 lentiviral vector (puro), and the targeting sequences are as follows (both in cDNA): p62 sgRNA#1, 5′-GAAGATGTCATCCTTCACGT, and p62 sgRNA#2, 5′-TTCGGATTCTGGCATCTGTA. Lentivirus packing and transfection and selection of stable polyclonal transfectants with puromycin were carried out as detailed in our previous publication (72). shRNA expression was induced by 1 μg/ml doxycycline.

### Transfection.

For transfection of BJAB, P3HR1, and DG75, Gene Pulser XCell (Bio-Rad) was used with optimized programs. These representative cell lines were chosen in that they are easier to be transfected with this technique, compared with other lymphoma cell lines. Virus-transformed cell lines (type 3 latency) were transfected with Nucleofector (Lonza) or with lentivirus-mediated transfection as detailed in our previous publications (30, 37, 72). 293 and 293T cells and MEFs were transfected with Fugene HD (Promega) and Lipofectamine (Invitrogen), respectively.

### Promoter-reporter assays.

293 cells were transfected with expression plasmids as indicated together with p62p-Luc2 (or its mutants) and *Renilla* luciferase as an internal transfection control. Empty vector was used to equalize the total amounts of DNA in all transfections. Cells were collected 24 h after transfection. Luciferase activity was measured with equal amounts (10% of the total for each sample) of protein lysates with the use of a dual-luciferase assay kit (Promega) on a multimode microplate reader (Turner Biosystems). Results are means and standard errors (SE) for duplicates for each sample. At least three consistent results were obtained from independent experiments, and representative results are shown. The ability of the empty vector controls to activate the promoter constructs was set to 1.

### Amnis imaging flow cytometry and image acquisition.

Cells were fixed in 4% paraformaldehyde (PFA) for 15 min, permeabilized with 0.1% Triton X-100 in phosphate-buffered saline (PBS) for 30 min, blocked with 1% bovine serum albumin (BSA) in PBS for 1 h, and then incubated with 1:50 mouse anti-p65 (Invitrogen) for 1 h. Cells were washed with PBS with 0.1% Tween 20 three times and then incubated with 1:300 anti-mouse Alexa 555 (Invitrogen). Nuclei were stained with DAPI (4′,6-diamidino-2-phenylindole; Thermo Fisher). p65 was visualized using channel 3 (500 to 560 nm), and DAPI was visualized using channel 7 (430 to 505 nm). Fifty thousand cells were collected for each group and photographed through the Amnis software.

Statistical and graphical analyses were carried out using IDEA software, by methods described elsewhere (47, 73, 74). Briefly, the two colors (p65 and DAPI) were taken to the same compensation matrix, which was created using raw image files of single-color controls, and 1,000 events were recorded. Individual groups were gated for focus and singly and doubly positive cells, using the nuclear wizard within the software.

### Immunoprecipitation and immunoblotting.

For endogenous proteins, 1 × 10^7^ cells for each sample were used. For overexpressed proteins, 293T cells in 60-mm dishes were transfected with a total of 2 μg plasmids, and cells were cultured for 2 days before assays. Cell lysates were lysed with NP-40 lysis buffer (150 mM NaCl, 1% NP-40, 50 mM Tris [pH 8.0], and protease inhibitors), followed by immunoprecipitation (IP) with 1.5 μg of the indicated antibodies for overnight, and then incubated with 40 μl protein A/G beads (Santa Cruz) for 1 h. For ubiquitination assays, denaturing IP was performed, as detailed in our previous publication (21). After extensive washes, proteins on beads were subjected to immunoblotting (IB) with indicated antibodies, and signals were detected with an enhanced chemiluminescence (ECL) kit following the manufacturer’s protocol (Amersham Pharmacia Biotech).

### RNA extraction and real-time quantitative PCR.

Total RNA was isolated from tested cells using an RNeasy minikit according to the manufacturer's protocols (Qiagen). Reverse transcription was performed with the use of a GoScript RT kit following the manufacturer's instructions (Promega). Quantitative real-time PCR (qPCR) was performed with the use of SYBR green (Applied Biosystems), on a CFX96 real-time PCR detection system (Bio-Rad). All reactions were run in triplicates. Mean cycle threshold (*C_T_*) values were normalized to 18s rRNA, yielding a normalized *C_T_* (Δ*C_T_*). ΔΔ*C_T_* value was calculated by subtracting respective control from the Δ*C_T_*, and the expression level was then calculated by 2 raised to the power of respective −ΔΔ*C_T_* value. The averages of 2^−ΔΔ^*^CT^* in the control samples were set to 1 or 100%. Results are the averages and SE for duplicates or triplicates for each sample. Primers for real-time qPCR are as follows: for p62, F, 5′-CAGGCGCACTACCGCGATG-3′, and R, 5′-ACACAAGTCGTAGTCTGGGCAGAC-3′; for Cox2, F, 5′-CGGTGAAACTCTGGCTAGACAG-3′, and R, 5′-GCAAACCGTAGATGCTCAGGGA-3′; for HO1, F, 5′-CCAGGCAGAGAATGCTGAGTTC-3′, and R, 5′-AAGACTGGGCTCTCCTTGTTGC-3′; for BIC, F, 5′-ACCAGAGACCTTACCTGTCACCTT-3′, and R, 5′-GGCATAAAGAATTTAAACCACAGATTT-3′; for Blimp1, F, 5′-ACACACGGGAGAAAAGCCAC-3′, and R, 5′-CTTGTGGCACTGGGAGCAC-3′; for Bcl6, F, 5′-CGCAACTCTGAAGAGCCACCTGCG-3′, and R, 5′-TTTGTGACGGAAATGCAGGTTA-3′; for 18S rRNA, F, 5′-GGCCCTGTAATTGGAATGAGTC-3′, and R, 5′-CCAAGATCCAACTACGAGCTT-3′.

### Algorithm-based analysis.

The algorithm platform Gene Expression Profiling Interactive Analysis (GEPIA v2; http://gepia2.cancer-pku.cn) (48) was used for Pearson correlation analysis of the Genotype-Tissue Expression (GTEx) data set, which includes 174 EBV-transformed LCLs. The analysis was carried out using the default settings, and the GAPDH RNA level was used for normalization of RNA levels of all genes. A *P* value of <0.05 was considered statistically significant, and a value of >0.05 was nonsignificant; a *P* value of <0.01 was considered statistically very significant.

### Apoptosis and proliferation assays.

Apoptosis was quantified using flow cytometry as detailed in our previous publication (37), for annexin V binding (BD Biosciences) and 7-aminoactinomycin D (7-AAD) expression (eBioscience). Caspase 3 activity was evaluated by immunoblotting. An MTT [3-(4,5-dimethyl-2-thiazolyl)-2,5-diphenyl-2H-tetrazolium bromide] proliferation assay was carried out following the manufacturer’s instructions (Promega).

### Statistical analysis.

Data are expressed as means and SE for duplicate or triplicate samples, and representative results from at least three independent repeats with similar results are shown. Unpaired, two-tailed Student *t* tests were executed using GraphPad Prism (version 6) to compare the experimental interventions and controls. *P* values of <0.05, <0.01, and <0.001 were considered significant.
